# Expression of Proinflammatory Cytokines Is Upregulated in the Hypothalamic Paraventricular Nucleus of Dahl Salt-Sensitive Hypertensive Rats

**DOI:** 10.3389/fphys.2018.00104

**Published:** 2018-02-22

**Authors:** Enshe Jiang, Andrew D. Chapp, Yuanyuan Fan, Robert A. Larson, Taija Hahka, Michael J. Huber, Jianqun Yan, Qing-Hui Chen, Zhiying Shan

**Affiliations:** ^1^Department of Kinesiology and Integrative Physiology, Michigan Technological University, Houghton, MI, United States; ^2^Institute for Nursing and Health Research, Henan University, Kaifeng, China; ^3^Department of Physiology and Pathophysiology, School of Basic Medical Sciences, Xi'an Jiaotong University Health Science Center, Xi'an, China

**Keywords:** paraventricular nucleus, hypertension, high salt diet, proinflammatory cytokines, cerebrospinal fluid

## Abstract

Accumulating evidence indicates that inflammation is implicated in hypertension. However, the role of brain proinflammatory cytokines (PICs) in salt sensitive hypertension remains to be determined. Thus, the objective of this study was to test the hypothesis that high salt (HS) diet increases PICs expression in the paraventricular nucleus (PVN) and leads to PVN neuronal activation. Eight-week-old male Dahl salt sensitive (Dahl S) rats, and age and sex matched normal Sprague Dawley (SD) rats were divided into two groups and fed with either a HS (4% NaCl) or normal salt (NS, 0.4% NaCl) diet for 5 consecutive weeks. HS diet induced hypertension and significantly increased cerebrospinal fluid (CSF) sodium concentration ([Na^+^]) in Dahl S rats, but not in normal SD rats. In addition, HS diet intake triggered increases in mRNA levels and immunoreactivities of PVN PICs including TNF-α, IL-6, and IL-1β, as well as Fra1, a chronic marker of neuronal activation, in Dahl S rats, but not in SD rats. Next, we investigated whether this increase in the expression of PVN PICs and Fra1 was induced by increased CSF [Na^+^]. Adult male SD rats were intracerebroventricular (ICV) infused with 8 μl of either hypertonic salt (4 μmol NaCl), mannitol (8 μmol, as osmolarity control), or isotonic salt (0.9% NaCl as vehicle control). Three hours following the ICV infusion, rats were euthanized and their PVN PICs expression was measured. The results showed that central administration of hypertonic saline in SD rats significantly increased the expression of PICs including TNF-α, IL-6, and IL-1β, as well as neuronal activation marker Fra1, compared to isotonic NaCl controls and osmolarity controls. Finally, we tested whether the increase in PICs expression occurred in neurons. Incubation of hypothalamic neurons with 10 mM NaCl in a culture medium for 6 h elicited significant increases in *TNF*-α, *IL-6*, and *Fra1* mRNA levels. These observations, coupled with the important role of PICs in modulating neuronal activity and stimulating vasopressin release, suggest that HS intake induces an inflammatory state in the PVN, which, may in turn, augments sympathetic nerve activity and vasopressin secretion, contributing to the development of salt sensitive hypertension.

## Introduction

Hypertension remains one of the most common diseases in the United States and it affects about 29% adult populations and more than 65% persons older than 60 years (Kovell et al., 2015). The sustained elevated blood pressure causes many systemic insults such as kidney disease (Townsend and Taler, 2015), cardiovascular disease and stroke (Angeli et al., 2014). While there are many contributing factors to hypertension including genetics and ethnicity, a major contributing factor to hypertension is diet (de Wardener et al., 2004). High dietary intake of sodium chloride (salt), especially prevalent in western cultures, has been identified as a major risk factor for the development of hypertension (de Wardener et al., 2004; Drenjancevic-Peric et al., 2011). Inflammation has been implicated in hypertension and cardiovascular disease. Increased circulating levels of pro-inflammatory cytokines (PICs) including Tumor necrosis factor alpha (TNF-α), Interleukin-6 (IL-6), and Interleukin-1 beta (IL-1β) have been noted in both human hypertension and animal models of hypertension (Dalekos et al., 1996, 1997; Liu et al., 1996). Increased PICs expression has also been observed in the brain of several hypertensive animal models including AngII induced hypertensive rats (Kang et al., 2009) and spontaneously hypertensive rats (Waki et al., 2008). Furthermore, injection of PICs such as TNF-α and IL-1β in the brain cardiovascular control areas of normal rats can dramatically increase blood pressure (Wei et al., 2015). Despite this, the connection between high salt diet and changes in PICs in salt sensitive hypertension (SSHTN) has not been well established, especially in the central nervous system.

Previous study demonstrated that salt sensitive rats such as spontaneously hypertensive rats and Dahl salt sensitive (Dahl S) rats have impaired blood brain barriers, and high salt (HS) diet can increase their sodium concentration ([Na^+^]) in the cerebrospinal fluid (CSF) compared to normal salt (NS) diet (Huang et al., 2004). This evidence, coupled with the important role of PICs in regulating neuronal activity (Shi et al., 2010) have led us to hypothesize that HS diet intake increases CSF [Na^+^], which subsequently increase PICs expression in the paraventricular nucleus (PVN), the excessive production of PICs in turn leads to PVN neuron activation, and eventually results in blood pressure elevation. We will test this hypothesis in this study.

We focused on the PVN because the PVN is a key brain area controlling sympathetic outflow and blood pressure (Guyenet, 2006; Ye et al., 2013; Zucker et al., 2014). The PVN neurons integrate signals and inputs from circumventricular organs and other cardiovascular-relevant brain regions and convey the information to the rostral ventrolateral medulla (RVLM) or directly to spinal cord, to control sympathetic outflow (Ferguson et al., 2008). In addition, PVN sympathetic efferent activity can be modulated by multiple factors such as AngII, glutamate and PICs (Osborn et al., 2007; Shi et al., 2010; Ye et al., 2013). The PVN also plays an important role in salt-sensing mechanisms (Orlov and Mongin, 2007). HS diet induced hypertension in Dahl S rats can be prevented by lesions of the PVN (Azar et al., 1981). Taken together, we believe that HS diet intake may induce an increase in the PVN PICs expression, the excessive PICs in turn increases sympathetic outflow and eventually results in hypertension.

## Materials and methods

### Animals

All adult male rats including Dahl S rats and Sprague Dawley (SD) rats used in this study were purchased from Charles River Laboratories (Wilmington, MA). All animals were housed in two rats per cage and kept on a 12:12-h light-dark cycle in a climate-controlled room. Rat chow and water were provided *ad libitum*. This study was carried out in strict accordance with the recommendations in the Guide for the Care and Use of Laboratory Animals of the National Institutes of Health. The protocol was approved by the Michigan Technological University Institutional Animal Care and Use Committee.

### Blood pressure measurement

Blood pressure of all rats was measured by tail cuff method using a non-invasive blood pressure system (CODA, Kent Scientific Corporation) following manufacture's instruction. Blood pressure of each rat was measured for 3 connective days prior to 5 weeks diet treatment and the average value was used as a baseline. Then blood pressure was measured twice a week after different diets were given. To get blood pressure value as accurate as possible, prior to baseline blood pressure measurement, each rat was restrained in tail cuff tube for 15 min/day and then subjected to tail cuff measurement procedures. This procedure was repeated for 5 consecutive days. It would make rats get accustomed to blood pressure measurement procedures and ease their stress. Then their baseline blood pressure was measured for 3 consecutive days.

### The CSF collection and CSF [Na^+^] and osmolarity measurement

Rats were anesthetized with isoflurane (2.0–3.5% in O_2_) and were positioned in a stereotaxic frame. The rat head was flexed downward at an angle of ~35° to expose the dorsal surface of the hindbrain. After extraction of the muscles lying above the dorsal edge of occipital bone, the triangle of the dorsal surface of the medulla will be exposed. The needle tip of 1 ml insulin syringe (27G) was slowly punched through the Dura, and CSF was drawn into an insulin syringe by slowly aspiration. About 100 ~150 μl CSF from each rat was collected. After finishing the collection, the CSF was transferred into a 1.5 ml centrifuge tube and stored in −80 freezer until used. The CSF [Na^+^] were measured with a Dionex ICS-2100 ion chromatograph system (Thermo Fisher Scientific), and CSF osmolarity was measured with VAPRO (Wescor Inc MODEL 5600) following manufacture's instruction.

### Preparation of neuronal cultures

Primary neuronal cultures were made from the hypothalamus containing the PVN of 1-day-old SD rats as described previously (Shan et al., 2008). Briefly, rats were euthanized, and their hypothalamus was immediately dissected. Neurons were dissociated from hypothalamic tissues and plated in poly-L-lysine coated culture dishes with DMEM medium (Thermo Fisher Scientific) supplemented with 10% horse serum and 1% penicillin and streptomycin antibiotics. They were incubated for 10–14 days in 5% CO_2_ incubator and then were used. Neuronal cultures contained >90% neurons (remaining cells were primarily astrocytes).

### mRNA levels measurement

mRNA levels of PICs and *Fra1* in the PVN and cultured hypothalamic neurons were assessed using real time PCR as detailed previously (Huber et al., 2017; Fan et al., 2018). Briefly, PVN tissues were punched from rat brains. RNA from PVN tissues and cultured neurons were isolated using RNeasy plus Mini kit (Qiagen, CA, USA) following the manufacturer's instructions. Real-time PCR was performed to measure mRNA levels of *TNF-*α, *IL-6, IL-1*β, and *Fra1* using the Step One Plus Real Time PCR System (Applied Biosystems). Taqman primers and probes for *TNF-a* (Rn99999017_m1), *IL-6* (Rn01410330_m1), *IL-1*β (Rn00580432_m1) and *Fra1* (Rn 00564119_m1) were purchased from Applied Biosystems (Foster City, CA). Data were normalized to GAPDH (Rn01775763_g1) mRNA.

### Intracerebroventricular (ICV) injections

The ICV infusion was performed as detailed in our recent publications (Huber et al., 2017; Fan et al., 2018). In brief, adult male SD rats (350–400 g) were anesthetized with isoflurane (3% in O_2_) and were placed in a stereotaxic head frame. Then the skull was leveled between bregma and lambda. A small piece of skull was removed so that a Hamilton syringe could be lowered vertically into the left lateral ventricle. The stereotaxic coordinates for ICV injection into the left lateral ventricle were: 0.8–0.9 mm caudal to bregma; 1.4–1.8 mm lateral to midline; and 3.2–3.8 mm ventral to dura. Each rat received only one injection. The volume of 8 μL of either hypertonic saline (4 μmol NaCl), or mannitol (8 μmol, osmolarity control) or isotonic saline (0.9% NaCl, as vehicle control) was injected at the rate of 1 μl/min into the lateral ventricle using a UltraMicroPump3 (WPI). Three hours following ICV injection, rats were euthanized and their brain PVN tissues were punched out and received real time PCR analysis of genes of interest.

### Immunoreactivity assessment of PICs and Fra1

Immunostaining of PVN TNF-α, IL-6, and Fra1 was carried out with the following protocols: brain coronal sections (25 μm) containing the PVN were first washed in PBS three times for 10 min each. After that, sections were incubated with 5% horse serum in PBS for 30 min, and then incubated with either mouse anti-TNF-α antibody (1:100 dilution, Cat#:SC-52746), or mouse anti-IL-6 antibody (1:100 dilution, Cat#:SC-32296), or mouse anti-Fra1antibody (1:100 dilution, Cat#:SC-28310) in PBS containing 0.5% Triton X-100 and 5% horse serum for 72 h at 4°C. Afterwards, brain sections were washed with PBS three times for 10 min each. Then they were incubated with secondary antibody Alexa Fluor 488 donkey anti-mouse IgG (1:000) or Alexa Fluor® 594 donkey anti-mouse IgG (1:1000) overnight at 4°C. To verify that the observed antibody staining is specific, negative control was performed for each antibody in each experiment. The brain sections used for negative control were subjected to identical protocols as regular immunostaining except that primary antibody was replaced with the same volume of PBS. This will ensure that the secondary antibody does not non-specifically bind to certain cellular compartments and generate fluorescence. The sections were mounted in Vectashield mounting medium and images were taken with a Leica DMIL microscope. Low magnification images (40X) were used for quantification of immunoreactivity of PICs and Fra1 as described in the below.

### Quantification of immunoreactivity of PICs and Fra1 within specific PVN subnuclei

The images compared to one another were acquired under identical conditions, then their brightness and contrast were adjusted in PowerPoint using identical settings. Immunoreactivities of PICs and Fra1 were quantified in both fluorescence intensity and density of positive cells in three PVN subnuclei including dorsal cap (DC), lateral magnocellular (LM), and ventromedial (VM) region (Swanson and Kuypers, 1980; Higa-Taniguchi et al., 2007) in the medial level of PVN (~1.8 to 2.1 mm caudal to the bregma). The Rat Brain in Stereotaxic Coordinates (Paxinos and Watson, 2007) was used as a reference. Fluorescence intensity (also named corrected total cell fluorescence, or CTCF) was measured using Image J (NIH, Bethesda, USA), and numbers of positive stained cells were counted using Image Pro Plus (Media Cybernetics, Silver Spring. MD) following the software programs' instructions. The density of positive cells was calculated using positive cell numbers normalized with the area of the target subnucleus. The region of interest in the PVN was first drawn within the DC, LM, and VM subnuclei before fluorescence intensity measurement and positive cell number counting. On average, 7–15 sections were analyzed for each protein in each subnucleus. We attempted to analyze 3 sections per animal and 5 rats in total. However, in some cases sections were damaged during the incubation procedure. Thus, the mean value of target protein' fluorescence intensity or density of positive cells per section per subnucleus region was calculated within each experimental group for quantitative purposes. The fluorescent intensity and the density of positive cells are expressed as relative units by assigning the mean values of CTCF or density of positive cells in DC subnucleus of the control group as arbitrary unit 1.

### Reagents and antibodies

Primary antibodies including mouse anti-TNF-α, mouse anti-IL-6, mouse anti-IL-1β and mouse anti-Fra1 were all purchased from Santa Cruz Biotechnology (Dallas TX, USA). Secondary antibodies donkey anti-mouse (H+L) Alexa Fluor® 488 and donkey anti-mouse IgG (H+L) Alexa Fluor® 594 were purchased from Thermo Fisher Scientific (Waltham, MA, USA). Vectashield mounting medium was purchased from Vector Labs (Burlingame, CA, USA).

### Data analysis

All data are expressed as mean ± SEM. The data were analyzed using GraphPad Prism 5.0 for Windows (GraphPad Software). Statistical significance was calculated by a two-tailed Student's unpaired *t*-test. Differences were considered statistical significance at a critical value of *P* < 0.05.

## Results

### HS intake induces increases in water intake and urine excretion in both dahl S rats and SD rats, but elevates CSF [Na^+^] and results in hypertension only in Dahl S rats

**Figure 1:**
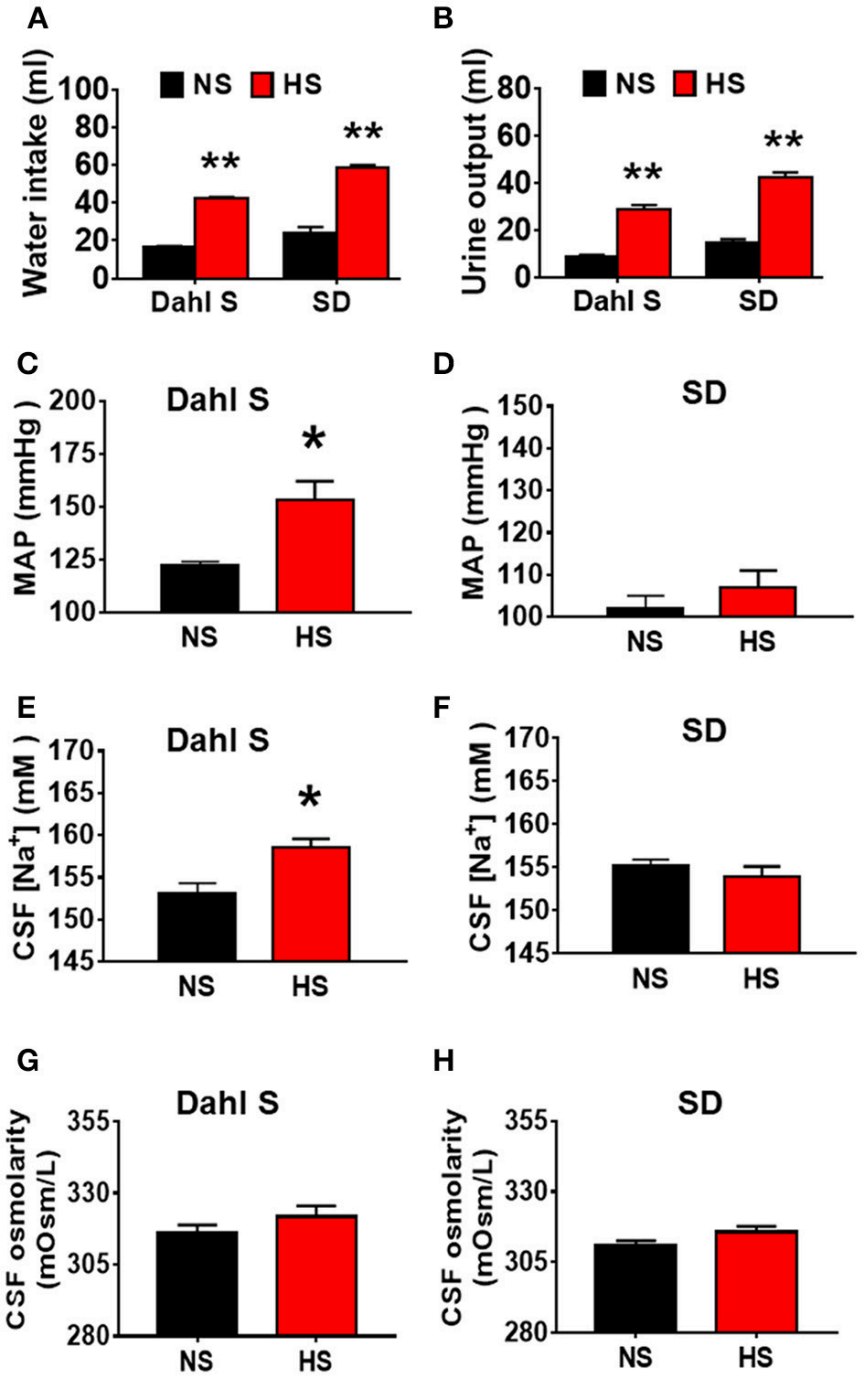
High salt (HS) intake induces an increase in water intake and urine output in both Dahl salt sensitive (Dahl S) rats and Sprague Dawley (SD) rats, but only increases mean arterial pressure (MAP) and cerebrospinal fluid (CSF) sodium concentration ([Na^+^]) in Dahl S rats. **(A,B)** show the summary data for water intake and urine output in Dahl S rats and SD rats with normal salt (NS) or HS diet, respectively. **(C,E,G)** show the changes in MAP, CSF [Na^+^], CSF osmolarity in Dahl S rats on different diets. **(D,F,H)** show the corresponding changes of MAP, CSF [Na^+^] and CSF osmolarity in SD rats. *n* = 5/group. ^*^P < 0.05; ^**^P < 0.01 compared with the rats on NS diet.

Our recent publication showed that HS intake can induce hypertension in Dahl S rats but not in normal SD rats (Huber et al., 2017). In this study, we aimed to test whether HS diet alters rats' metabolism, and whether the increased blood pressure was correlated with alteration in metabolism, CSF [Na^+^] and CSF osmolarity. Eight-week-old male Dahl S rats and age and sex matched SD rats were divided into two groups and were fed either an NS (0.4% NaCl) or HS (4% NaCl) diet. Their blood pressure was measured via tail cuff method. Four and half weeks following different diet treatment, all rats were transferred into individual metabolic cages, and allowed 48 h to get used to the environment, then their basal 24 h water intake, urine excretion were assessed. Rats then were euthanized, and their CSF were collected for [Na^+^] and osmolarity assessment. HS diet intake dramatically increased water intake and urine execration in both Dahl S rats (water intake, NS: 16.7 ± 0.5 vs. HS: 42.5 ± 0.6 ml; urine output, NS: 9.0 ± 0.7 vs. HS: 29.0 ± 1.7 ml) and SD rats (Water intake, NS: 23.9 ± 3.3 vs. HS: 58.4 ± 1.4 ml; urine output, NS: 14.5 ± 1.7 vs. HS: 42.6 ± 2.0 ml) compared to their NS intake controls (Figures 1A,B) In addition, in Dahl S rats, HS intake significantly increased mean arterial pressure (MAP) (NS: 122 ± 2 vs. HS: 153 ± 9 mmHg, *P* < 0.05), and CSF [Na^+^] (NS: 153.0 ± 1.3 vs. HS: 158.5 ± 1.1 mM, *P* < 0.05), but not significantly altered CSF osmolarity (NS: 316.3 ± 2.6 vs. HS: 321.8 ± 3.8 mOsm/L, *P* > 0.05) compared to Dahl S rats on a NS diet (Figures 1C,E,G). These changes in blood pressure and CSF [Na^+^] were specific to Dahl S rats as normal SD rats showed no significant changes in MAP (NS: 102 ± 3 vs. HS:107 ± 4 mmHg, *P* > 0.05), CSF [Na^+^] (NS: 155.2 ± 0.7 vs. HS: 153.9 ± 1.2 mM, *P* > 0.05), CSF osmolarity (NS: 310.8 ± 1.8 vs. HS: 315.8 ± 1.9 mOsm/L, *P* > 0.05) (Figures 1D,F,H). These results suggest that alteration of metabolism is not a major factor resulting in hypertension since normal SD rats also increased water intake and urine excretion in response to HS diet but keep normal blood pressure. Thus, high sodium intake induced increase in CSF [Na^+^] maybe a major contributing factor to elevated MAP in Dahl S rats.

### HS diet intake increases PICs expression in the PVN of Dahl S rats

**Figure 2:**
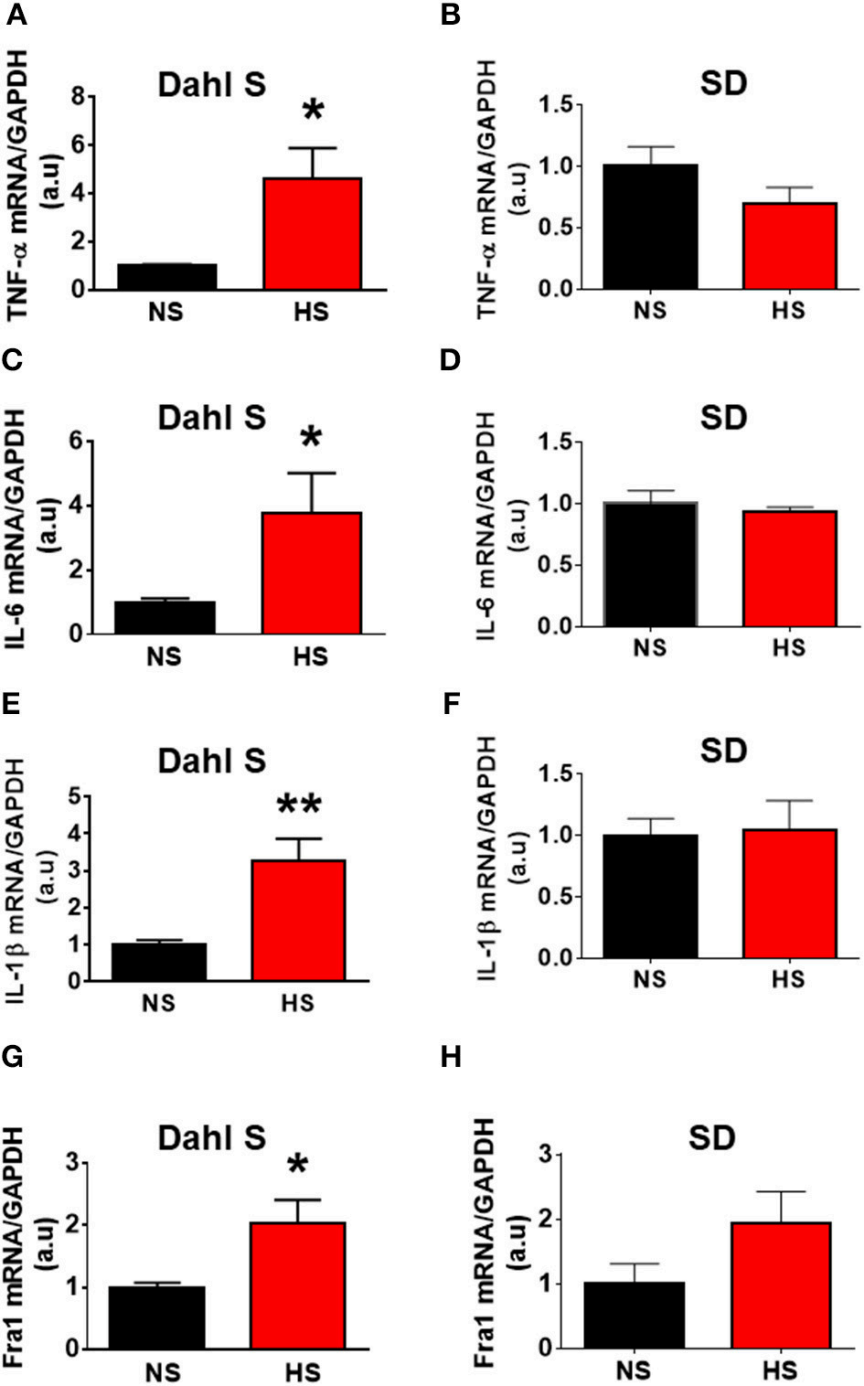
High salt (HS) diet increased mRNA levels of proinflammatory cytokines (PICs) in the paraventricular nucleus of Dahl salt sensitive (Dahl S) rats but not in Sprague Dawley (SD) rats. Summary data show the changes of mRNA levels of *TNF-*α **(A)**, *IL-6* **(C)**, *IL-1*β **(E)**, and *Fra1* **(G)** in paraventricular nucleus of Dahl S rats on HS diet (red) and normal salt (NS) diet (black). **(B,D,F,H)** show the corresponding changes of PICs in Sprague Dawley rats. *n* = 5/group. ^*^P < 0.05; ^**^P < 0.01.

In this study, we first tested whether HS diet intake can alter PVN PICs mRNA levels in Dahl S rats and normal SD rats. Eight-week-old male Dahl S rats and age and sex matched SD rats were divided into two groups and were fed either an HS or NS diet for 5 weeks. The rats were then euthanized, their PVN tissues were punched out, and real time PCR was performed to assess PICs mRNA levels using Taqman primers and probes. The results indicated that HS intake dramatically increased the mRNA levels of PICs including *TNF-*α (NS: 1.00 ± 0.09 vs. HS: 4.58 ± 1.31, *P* < 0.05), *IL-6* (NS: 1.00 ± 0.12 vs. HS: 3.77 ± 1.24, *P* < 0.05), *IL-1*β (NS: 1.00 ± 0.12 vs. HS: 3.26 ± 0.60, *P* < 0.01) and *Fra1* (NS: 1.00 ± 0.07 vs. HS: 2.03 ± 0.38, *P* < 0.05) in Dahl S rats (Figures 2A,C,E,G). In contrast, HS diet did not alter the mRNA expressions in the PVN PICs including *TNF-*α (NS: 1.00 ± 0.16 vs. HS: 0.69 ± 0.14, *P* > 0.05), *IL-6* (NS: 1.00 ± 0.11 vs. HS: 0.93 ± 0.04, *P* > 0.05), and *IL-1*β (NS: 1.00 ± 0.14 vs. HS: 1.04 ± 0.24, *P* > 0.05), as well as neuronal activation marker, *Fra1* (NS: 1.00 ± 0.31 vs. HS: 1.93 ± 0.50, *P* > 0.05) in normal SD rats (Figures 2B,D,F,H).

**Figure 3:**
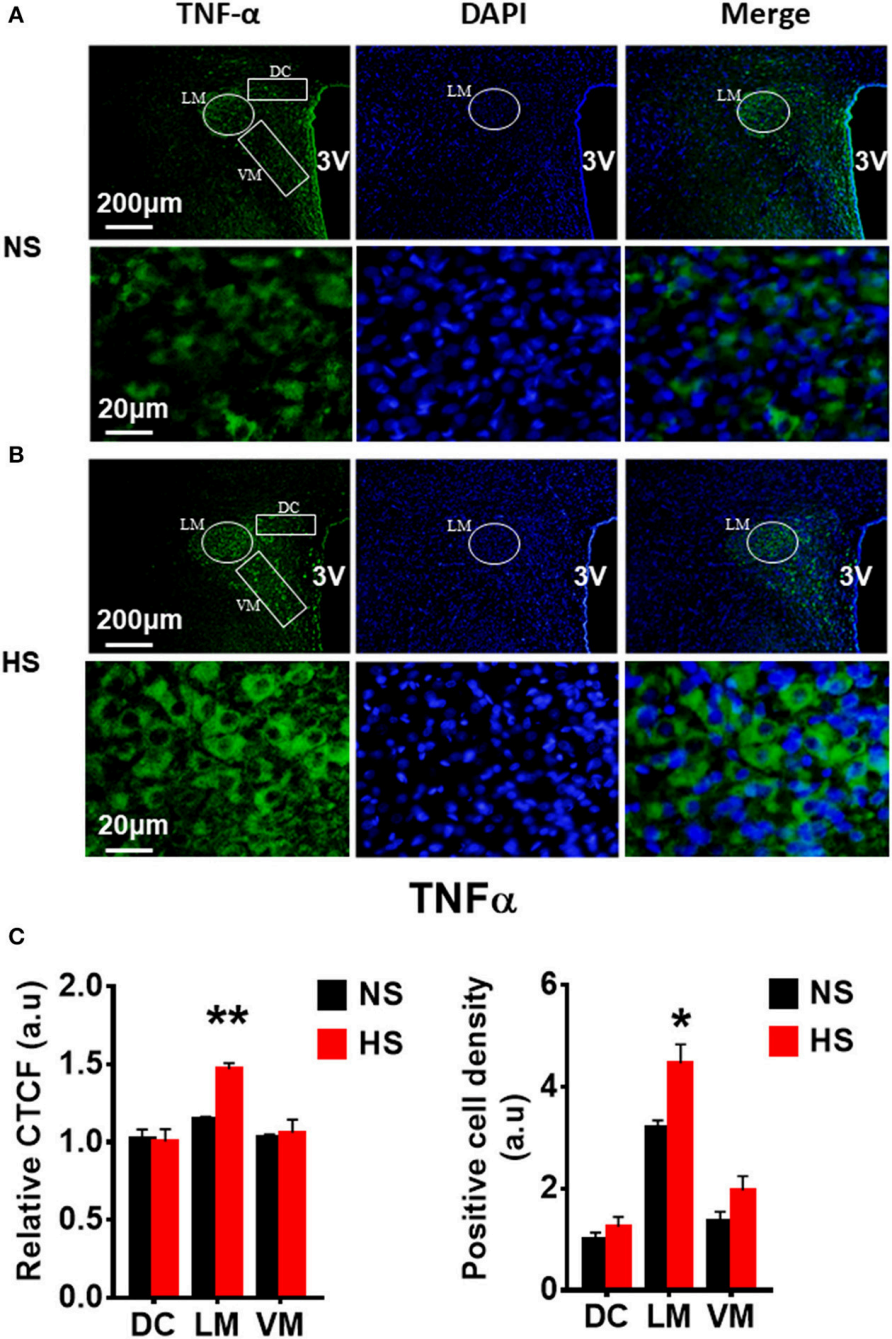
High salt intake increased TNF-α protein expression in the paraventricular nucleus (PVN) of Dahl salt sensitive (Dahl S) rats. Representative images showing immunostaining of TNF-α (green), DAPI (blue) and merged image in PVN from a Dahl S rat on normal salt (NS) diet **(A)** and a Dahl S rat on HS diet **(B)**. The area boxed in circle in upper panel was higher magnified in the lower panel. The brain coronal sections were taken from ~1.8–2.1 mm caudal to the bregma. Brightness and contrast were adjusted in the PowerPoint software to the same settings for all images. **(C)** Summary data showing the relative CTCF and positive cell density of TNF-α protein expression within identified PVN subnuclei in the 2 groups (*n* = 5/group). 3V, the third ventricle; DAPI, 4′,6-diamidino-2-phenylindole dihydrochloride; CTCF, the corrected total cell fluorescence. ^*^P < 0.05; ^**^P < 0.01.

**Figure 4:**
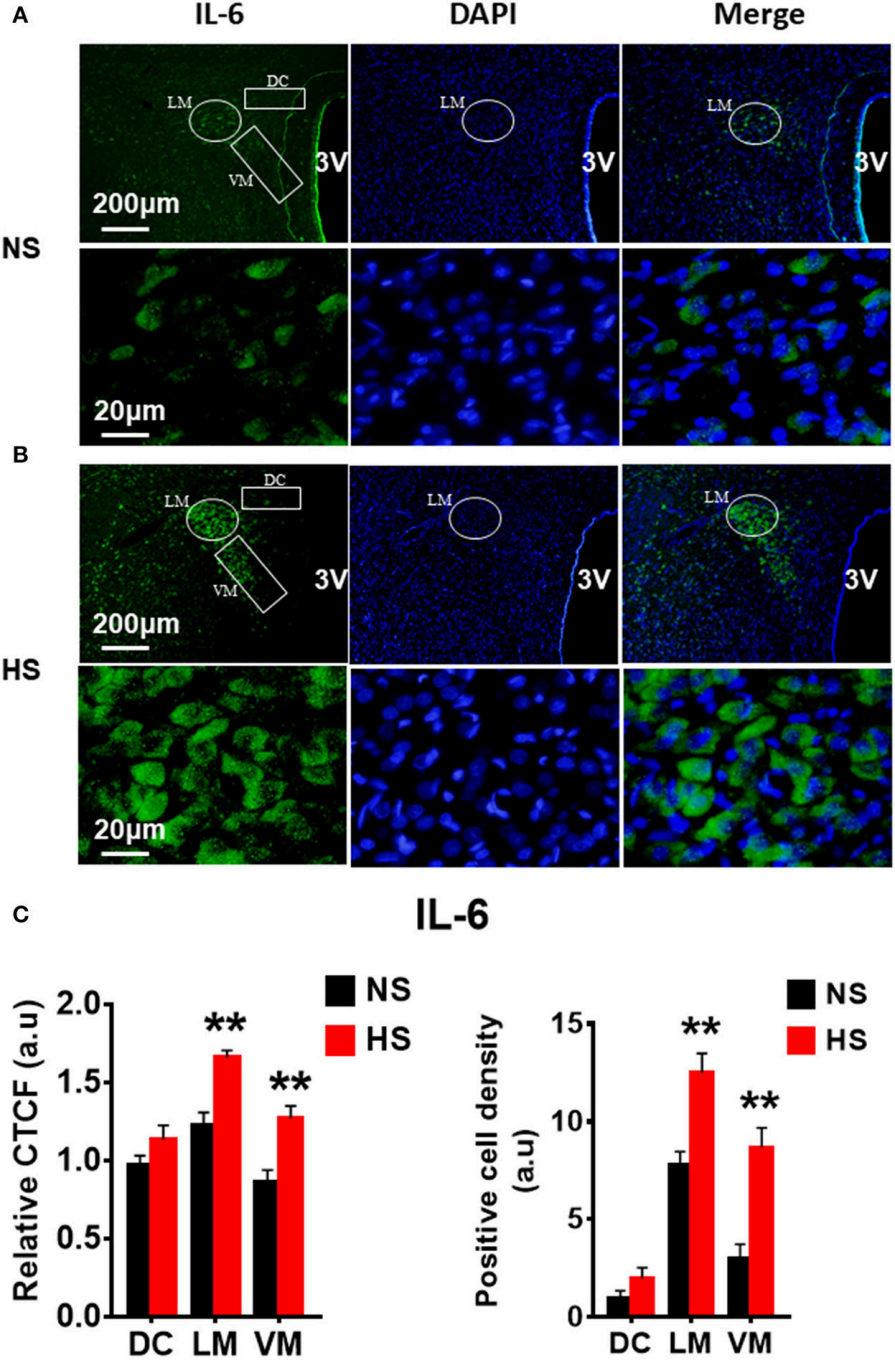
High salt intake increased IL-6 protein expression in the paraventricular nucleus (PVN) of Dahl salt sensitive (Dahl S) rats. Representative images showing immunostaining of IL-6 (green), DAPI (blue) and merged image in PVN from a Dahl S rat on normal salt (NS) diet **(A)** and a Dahl S rat on HS diet **(B)**. The area boxed in circle in upper panel was higher magnified in the lower panel. The brain coronal sections were taken from ~1.8 to 2.1 mm caudal to the bregma. Brightness and contrast were adjusted in the PowerPoint software to the same settings for all images. **(C)** Summary data showing the relative CTCF and positive cell density of IL-6 protein expression within identified PVN subnuclei in the 2 groups (*n* = 5/group). 3V, the third ventricle; DAPI, 4′,6-diamidino-2-phenylindole; CTCF, the corrected total cell fluorescence. ^**^P < 0.01.

**Figure 5:**
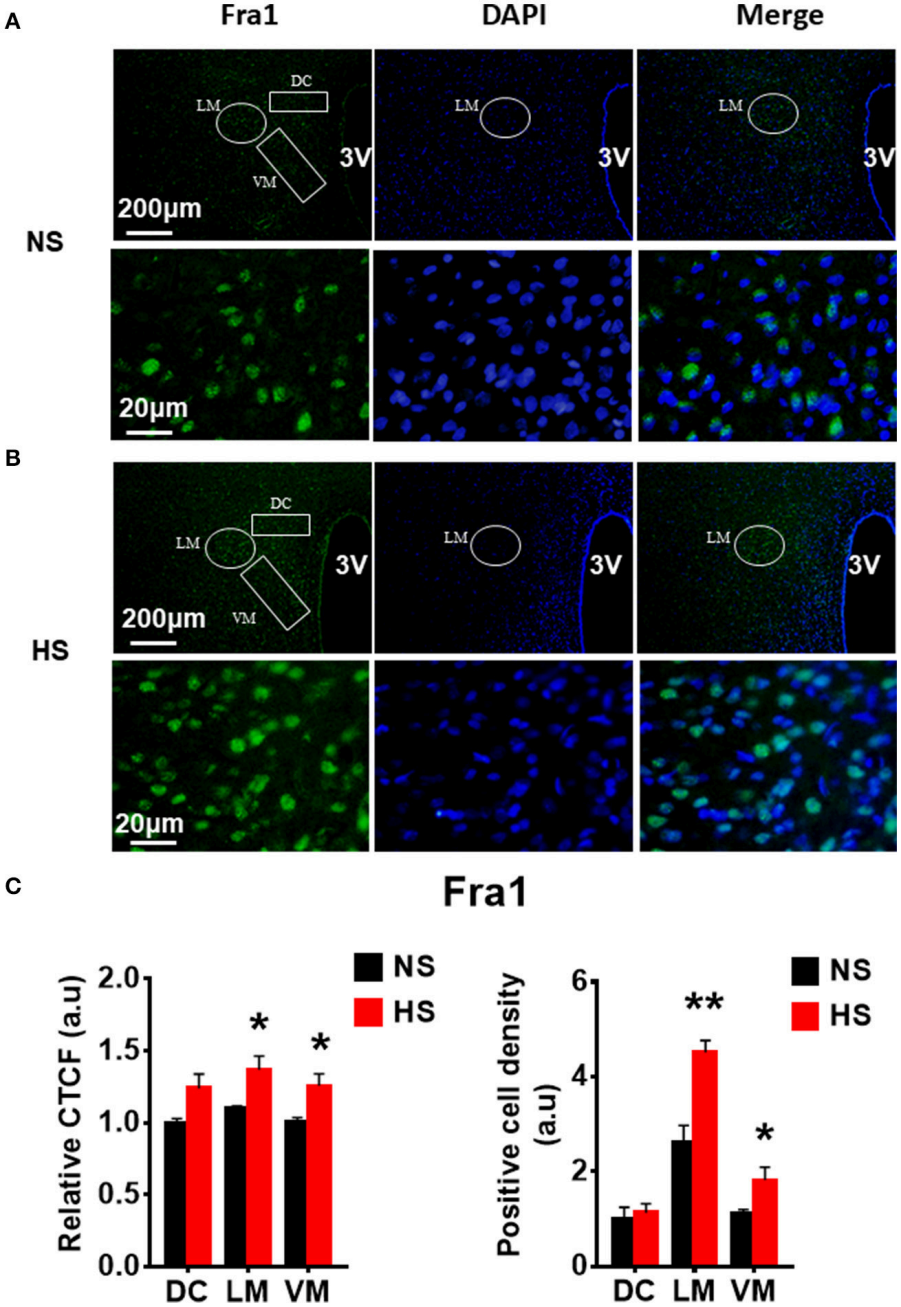
High salt intake increased Fra1 protein expression in the paraventricular nucleus (PVN) of Dahl salt sensitive (Dahl S) rats. Representative images showing immunostaining of Fra1 (green), DAPI (blue) and merged image in PVN from a Dahl S rat on a normal salt (NS) diet **(A)** and a Dahl S rat on a HS diet **(B)**. The area boxed in circle in upper panel was higher magnified in the lower panel. The brain coronal sections were taken from ~1.8 to 2.1 mm caudal to the bregma. Brightness and contrast were adjusted in the PowerPoint software to the same settings for all images. **(C)** Summary data showing the relative CTCF and positive cell density of Fra1 protein expression within identified PVN subnuclei in the 2 groups (*n* = 5/group). 3V, the third ventricle; DAPI, 4,6-diamidino-2-phenylindole; CTCF, the corrected total cell fluorescence. ^*^P < 0.05; ^**^P < 0.01.

We then determined whether HS diet also induces an increase in protein expression of PVN PICs and Fra1 in Dahl S rats. If yes, in which PVN subnucleus does this increase occurs. Additional 5 rats from each group were transcardially perfused with 4% paraformaldehyde (PFA), their brain coronal sections containing the PVN were cut and immunostainings of PICs and Fra1 were performed. Then fluorescence intensity (indicated by CTCF), and numbers of immunoreactive cells of anti-TNF-α, IL6 or Fra1 were measured, respectively, in three PVN subnuclei including dorsal cap (DC), ventromedial (VM) subnucleus, two major autonomic-related PVN subnuclei, as well as lateral magnocellular (LM) subnucleus, a region containing magnocellular neurosecretory neurons producing vasopressin and/or oxytocin. The results showed both stronger CTCF and increased positive cell density for TNF-α in LM (Figure 3), IL-6 in LM and VM (Figure 4), and Fra1 in LM and VM (Figure 5) regions, in Dahl S rats with HS diet compared to their cohort rats with NS diet. IL1-β immunostaining was also carried out but failed to detect its expression in Dahl S rats under any conditions. We believe this is due to the antibody. Thus, elevated dietary salt intake in Dahl S rats is capable of increasing PICs and neuronal activity in the PVN LM and VM subnuclei, while HS diet in non-diseased SD rats does not alter PVN PICs expression and neuronal activity.

### ICV infusion of hypertonic saline increases expression of PICs in the PVN of SD rats

**Figure 6:**
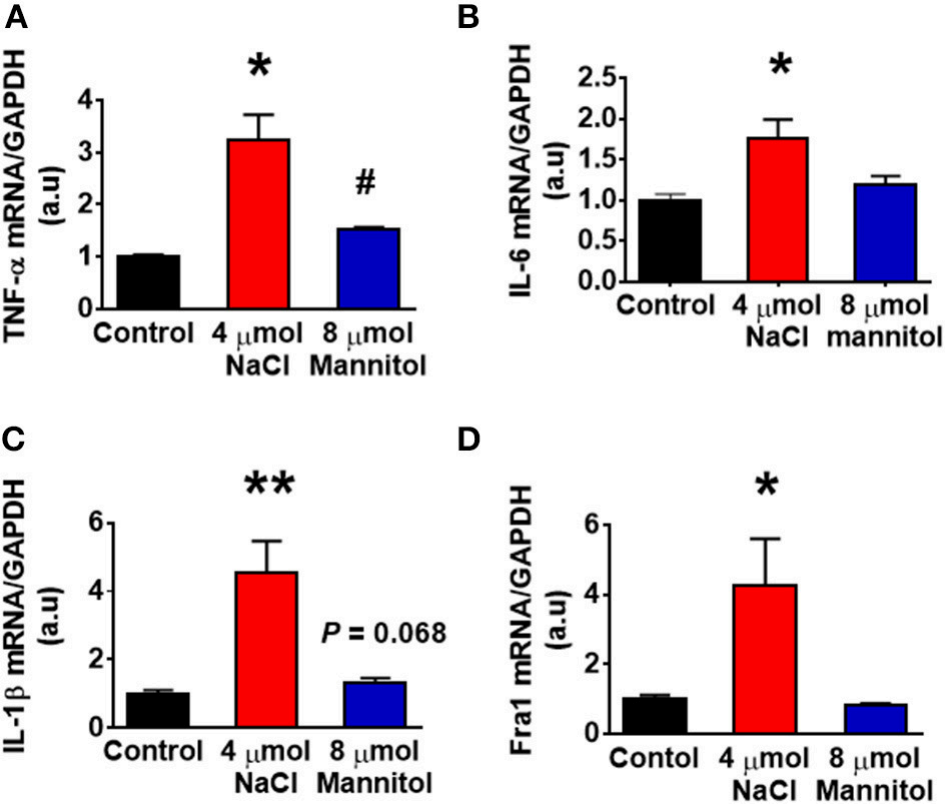
Intracerebroventricular (ICV) infusion of hypertonic saline increased mRNA levels of pro-inflammatory cytokines and *Fra1* in the paraventricular nucleus (PVN) of Sprague Dawley (SD) rats. SD rats (*n* = 5/group) were ICV infused with same volume (8 μl) of either isotonic saline (vehicle control, black), 4 μmol NaCl (red), or 8 μmol mannitol (osmolarity control, blue), their PVN tissues were punched out and analyzed for mRNA levels of *TNF-*α **(A)**, *IL-6* **(B)**, *IL-1*β **(C)**, and *Fra1* **(D)**. ^*^P < 0.05; ^**^P < 0.01, 4 μmol NaCl vs. Control or 8 μmol mannitol; ^#^ *P* < 0.05, 8 μmol mannitol vs. Control.

Figure 1 showed that HS intake increased CSF [Na^+^] in Dahl S rats. Figure 1 also showed an increasing trend in CSF osmolarity in Dahl S strain although statistical significance was not reached. To test if increases in brain sodium chloride elevated PICs expression and neuronal activity, and whether this increase is due to osmolarity changes. SD rats were ICV infused with 8 μl of either hypertonic saline (4 μmol NaCl), osmolarity control (8 μmol mannitol), or same volume of isotonic saline (0.9% NaCl) as vehicle control. Three hours following infusion, 5 rats from each group were euthanized, the PVN tissues were punched out and real time PCR was performed to measure the mRNA levels of PICs including *TNF-*α, *IL-6*, and IL-1β, as well as *Fra1*. Assuming the entire CSF volume of a SD rat is 400 μl (Tomiyama et al., 1999), ICV infusion of 4 μmol NaCl should increase the CSF [Na^+^] by 10 mM while our chronic study showed that 5-week of 4% HS diet increased CSF [Na^+^] by 5.5 mM in Dahl S rats (Figure 1E). In this acute study, we intentionally chose to double the increase in the CSF [Na^+^] to mimic the possible long term physiological effect of high salt diet induced increase in brain [Na^+^] on brain gene expression. The results showed that the mRNA levels of *TNF-*α (Control: 1.00 ± 0.04 vs. 4 μmol NaCl: 3.25 ± 0.47, *P* < 0.01), *IL-6* (Control: 1.00 ± 0.08 vs. 4 μmol NaCl: 1.76 ± 0.23, *P* < 0.05), *IL-1*β (Control: 1.00 ± 0.11 vs. 4 μmol NaCl: 4.53 ± 0.94, *P* < 0.01) and *Fra1* (Control: 1.00 ± 0.11 vs. 4 μmol NaCl: 4.24 ± 1.37, *P* < 0.05) were significantly elevated in hypertonic saline infusion rats compared to vehicle control rats. In addition, ICV infusion of 8 μmol mannitol, an osmolarity control, also induced mild but significant increase in the mRNA levels of *TNF-*α (1.5-fold), but no significant alterations were observed in the mRNA levels of *IL-6, IL-1*β, and *Fra1* compared to vehicle control (Figure 6). Thus, increases in the mRNA levels of PICs and *Fra1* were primarily attributed to the increases in CSF [Na^+^], and they were not primarily due to increases in CSF osmolarity, as mannitol had slightly or no effect on the increases in their mRNA levels.

**Figure 7:**
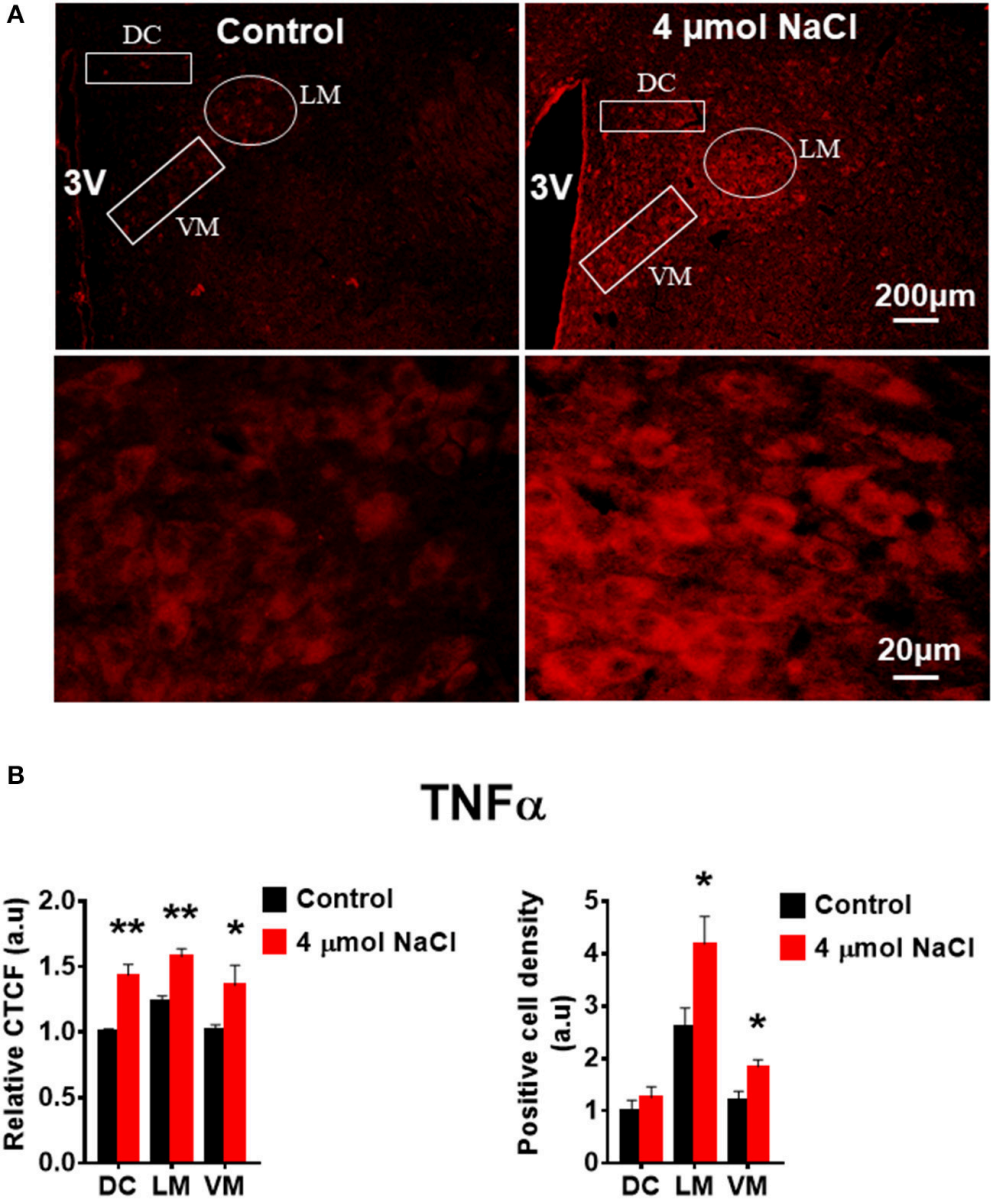
Intracerebroventricular (ICV) infusion of hypertonic saline increases immunoreactivity of TNF-α in the paraventricular nucleus (PVN) of Sprague Dawley (SD) rats. **(A)** Representative images show the TNF-α immunostaining from a SD rat that received isotonic saline (Control) and 4 μmol NaCl ICV infusion, respectively. The area boxed in circle in upper panel was higher magnified in the lower panel. The brain coronal sections were taken from ~1.8–2.1 mm caudal to the bregma. Brightness and contrast were adjusted in the PowerPoint software to the same settings for all images. **(B)** Summary data showing the relative CTCF and positive cell density of TNFα protein expression within identified PVN subnuclei in the 2 groups (*n* = 5/group). 3V, third ventricle; CTCF, the corrected total cell fluorescence. ^*^P < 0.05, ^**^P < 0.01 compared to Control.

**Figure 8:**
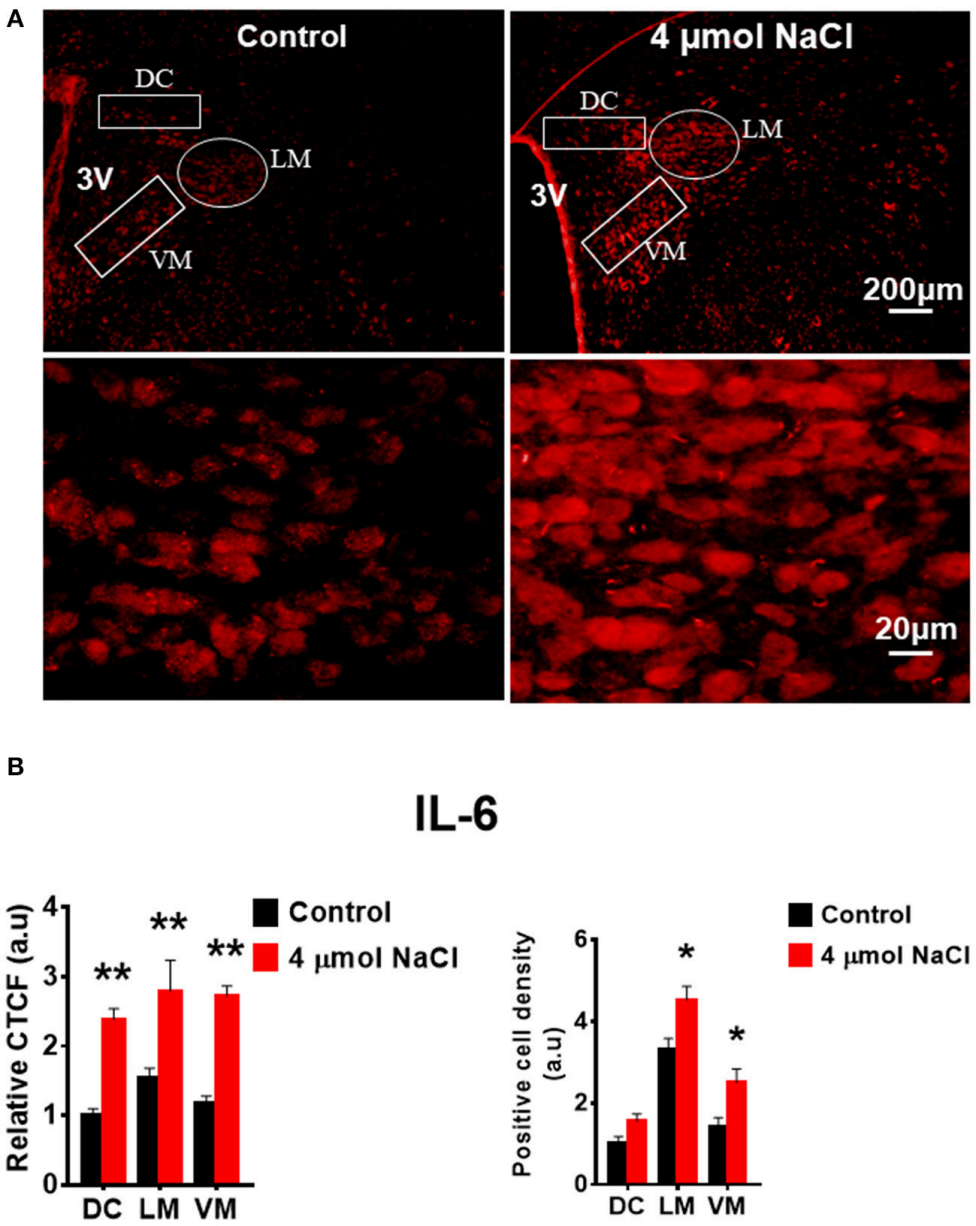
Intracerebroventricular (ICV) infusion of hypertonic saline increases immunoreactivity of IL-6 in the paraventricular nucleus (PVN) of Sprague Dawley (SD) rats. **(A)** Representative images show the IL-6 immunostaining from a SD rat that received isotonic saline (Control) and 4 μmol NaCl ICV infusion, respectively. The area boxed in circle in upper panel was higher magnified in the lower panel. The brain coronal sections were taken from ~1.8–2.1 mm caudal to the bregma. Brightness and contrast were adjusted in the PowerPoint software to the same settings for all images. **(B)** Summary data showing the relative CTCF and positive cell density of IL-6 protein expression within identified PVN subnuclei in the 2 groups (*n* = 5/group). 3V, third ventricle; CTCF, the corrected total cell fluorescence. ^*^P < 0.05, ^**^P < 0.01 compared to Control.

**Figure 9:**
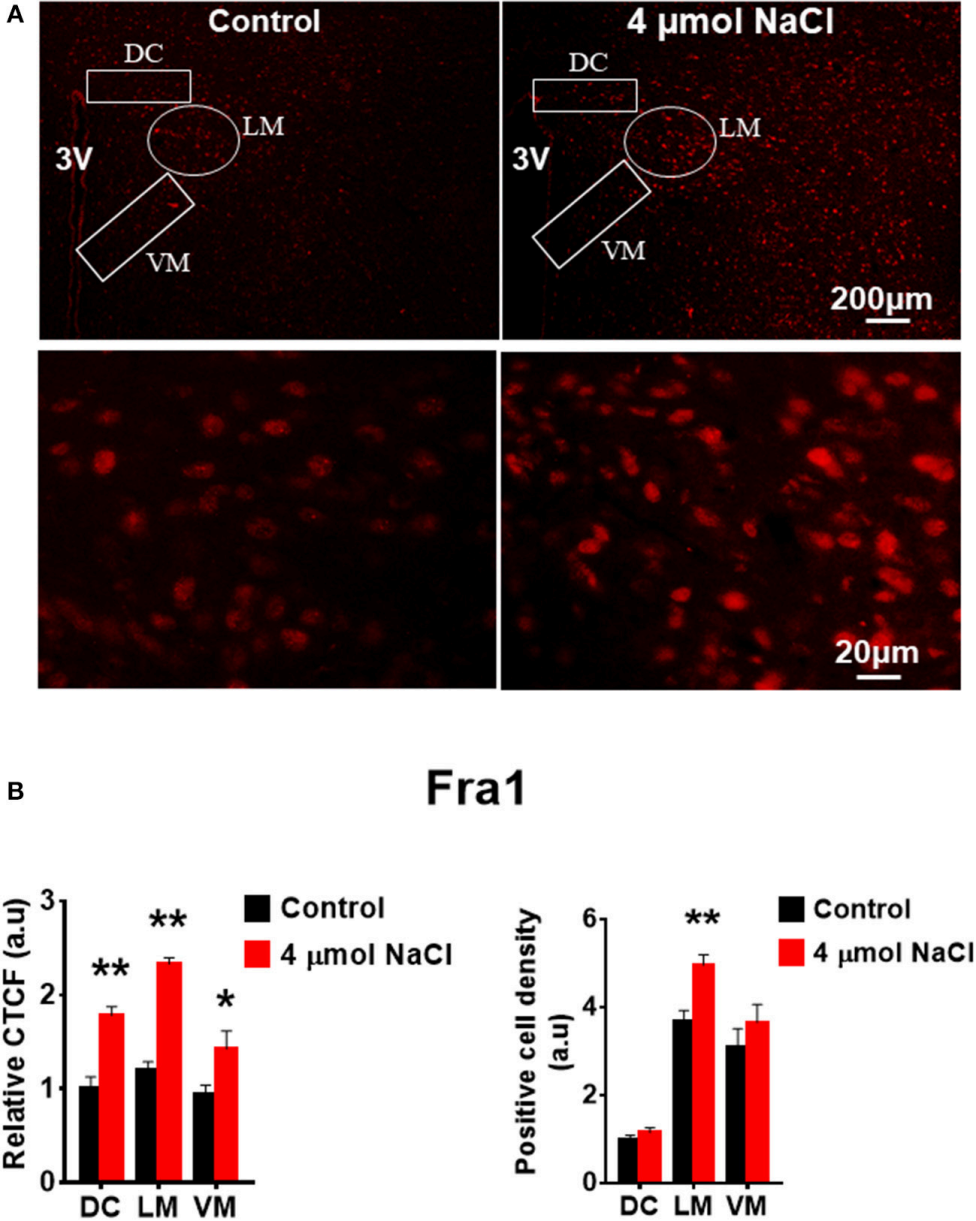
Intracerebroventricular (ICV) infusion of hypertonic saline increases immunoreactivity of Fra1 in paraventricular nucleus (PVN) of Spraque Dawley (SD) rats. **(A)** Representative images show the Fra1 immunostaining from SD rats that received isotonic saline (Control) and 4 μmol NaCl ICV infusion, respectively. The area boxed in circle in upper panel was higher magnified in the lower panel. The brain coronal sections were taken from ~1.8–2.1 mm caudal to the bregma. Brightness and contrast were adjusted in the PowerPoint software to the same settings for all images. **(B)** Summary data showing the relative CTCF and positive cell density of Fra1 protein expression within identified PVN subnuclei in the 2 groups (*n* = 5/group). 3V, third ventricle; CTCF, the corrected total cell fluorescence. ^*^P < 0.05, ^**^P < 0.01 compared to Control.

Additional 5 rats from each group were transcardially perfused with 4% PFA, and their brain coronal sections containing the PVN were cut and immunostainings of PICs and Fra1 were performed. Immunofluorescence intensity (indicated by CTCF), and positive cell density of each protein were measured, respectively, in the three subnuclei in the medial PVN. The results showed that stronger CTCF of TNF-α, IL6 and Fra1 occurred in all three PVN subnuclei including DC, LM and VM region in hypertonic saline injected rats compared to control rats (Figures 7–9). Increased positive cell density for TNF-α in LM and VM, IL-6 in LM and VM, and Fra1 in LM, were observed, in hypertonic salt injection rats compared to isotonic saline injected controls (Figures 7–9). Thus, increases in CSF [Na^+^] contribute to increases in expressions of PICs and Fra1, a chronic neuronal activation marker.

### Hypertonic saline treatment elicited an increase in PICs mRNA levels in hypothalamic neuronal cultures of neonatal SD rats

**Figure 10:**
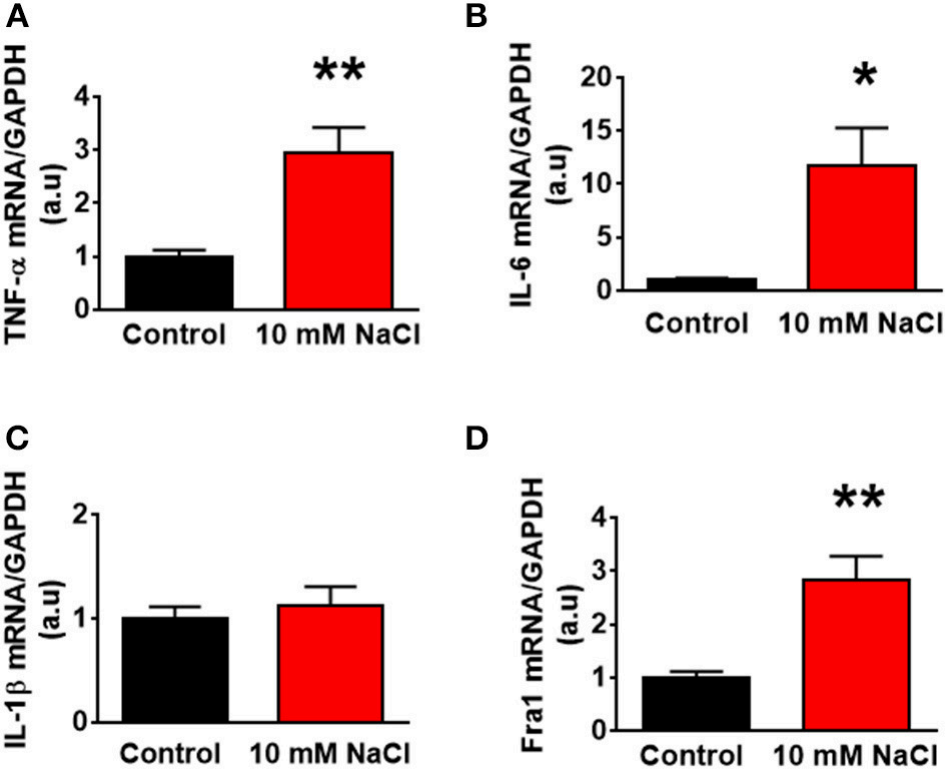
The changes of mRNA levels of proinflammatory cytokines treated with 10 mM NaCl. The summary data show the change of mRNA levels of *TNF-*α **(A)**, *IL-6* **(B)**, *IL-1*β **(C)**, and *Fra1* **(D)** in primary cultured paraventricular nucleus neurons treated with vehicle control (Control, black) or 10 mM NaCl (red). *n* = 5/group. ^*^P < 0.05; ^**^P < 0.01, 10 mM NaCl vs. Control.

In this experiment, we aimed to determine whether HS induced increase in PICs expression occur in brain neurons. We increased NaCl concentration by 10 mM in full culture medium to mimic the physiological effect of central administration of hypertonic salt on PVN gene expression. Neuronal cultures were prepared from the hypothalamus containing the PVN of neonatal SD rats, and cultured in DMEM medium supplement with 10% horse serum. Ten to fourteen days old neuronal cultures were used in this experiment. Incubation of neurons with 10 mM NaCl in cultured medium for 6 h elicited significant increases in the mRNA levels of *TNF-*α (Control: 1.00 ± 0.12 vs. 10 mM NaCl: 2.95 ± 0,48, *P* < 0.01), *IL-6* (Control: 1.00 ± 0.17 vs. 10 mM NaCl: 11.82 ± 3.46, *P* < 0.05) and *Fra1* (Control: 1.00 ± 0.11 vs. 10 mM NaCl: 2.84 ± 0.44, *P* < 0.01) compared to neurons treated with vehicle control in cultured medium. No significant difference in *IL-1*β mRNA expression was observed between the two groups. (Figure 10).

## Discussion

The present study reports three novel findings: (i) HS diet intake increases brain sodium concentration, and upregulates expression of PICs including TNF-α, IL-1β, and IL-6; as well as Fra1, a chronic neuronal activation marker, in the PVN of Dahl S rats but not normal SD rats; (ii) central administration of hypertonic saline elevates expression of PVN PICs and Fra1 in normal SD rats; and (iii) HS treatment stimulates *TNF-*α, *IL-6*, and *Fra1* expression in hypothalamic neurons from neonatal SD rats. The PVN is a key brain area controlling sympathetic outflow, blood pressure, and salt sensing mechanisms. The PVN also contains magnocellular neurons and can secrete peptide hormones such as arginine vasopressin (AVP) into circulation. Increased sympathetic nerve activity and upregulated plasma AVP have been demonstrated to play critical roles in the development and maintenance of SSHTN (Eslami and Tuck, 2003; Bardgett et al., 2014). The PICs are well known neuronal activity modulators (Kang et al., 2010; Shi et al., 2010) and AVP secretion stimuli (Yasin et al., 1994; Chikanza et al., 2000). Our findings suggest increases in the PVN PICs expression may contribute to the initiation and development of SSHTN.

The Dahl S rat is a common disease model for SSHTN (Somova et al., 1999). Multiple abnormalities have been identified in this strain of rats including increased blood brain barrier permeability (Pelisch et al., 2011); upregulated CSF [Na^+^] (Huang et al., 2004); increased plasma AVP (Serino et al., 2001); increased vascular inflammation (Viel et al., 2010); dysfunctional baroreflex control system (Bugenhagen et al., 2010); and the development of hypertension; following high dietary salt intake (Simchon et al., 1989). However, the underlying mechanism resting in these abnormalities is not clear. In this study, we first assessed whether metabolism is altered in response to HS diet intake, and whether metabolism alteration is correlated with blood pressure elevation in Dahl S rats. Age and sex matched normal SD rats were used as controls. The results showed that HS intake increases water intake and urine output in both Dahl S rats and SD rats compared to their normal diet intake controls (Figure 1A). While hypertension development only occurred in Dahl S rats (Figure 1B). These results suggest that alteration of metabolism is not responsible for hypertension development. We further measured osmolarity and [Na^+^] in CSF samples from Dahl S rats and SD rats with either a NS diet or HS diet. To be consistent with previous findings, our study showed that Dahl S rats on a high sodium diet increased CSF [Na^+^]. SD rats on high salt and normal salt diet had little or no change in CSF [Na^+^]. Previous studies indicated that high dietary salt intake can increase the concentration of plasma sodium in hypertensive and normotensive individuals (He et al., 2005). This suggests that peripheral changes in [Na^+^] from increased salt intake are unable to affect CNS [Na^+^] in normal SD rats and cause increases in MAP. Additionally, Dahl S rats on NS diet had substantially lower MAP and CSF [Na^+^] compared to the Dahl S rats on HS diet. Taken together, our results suggest that increased CSF [Na+] maybe a major driver resulting in SSHTN in Dahl S rats.

SSHTN is believed to be at least in part, neurogenic in nature. Increased sympathetic outflow has been observed in both human and animal models of SSHTN (Fujita et al., 1980; Gao et al., 2017). SSHTN is also characterized by increased AVP levels in the circulation of Dahl S rats with high salt diet (Serino et al., 2001). AVP can stimulate water reabsorption back into the circulation from the kidneys and constrict arterioles therefore increasing blood pressure. The hypothalamic PVN plays an important role in controlling both sympathetic outflow, and AVP synthesis and secretion. The PVN is a complex nucleus and is anatomically divided into several magnocellular and parvocellular subnuclei (Swanson and Kuypers, 1980), comprising functionally distinct neural populations (Cruz et al., 2008). The PVN pre-autonomic neurons, which primary distributed in the VM, DC, and parvocellular posterior subnuclei, send long descending projections to the RVLM (Chen and Toney, 2010) and/or intermediolateral cell column of the spinal cord (Zhou et al., 2015) therefore regulating sympathetic outflow. The PVN AVP producing neurons, which concentrated in the LM subnucleus, send axon projections to the posterior pituitary, where AVP are stored in secretory vesicles and released into peripheral circulation upon activation (Meyer-Lindenberg et al., 2011). AVP is released in response to many stimuli such as high osmolarity, stress and inflammatory signals (Chikanza et al., 2000; Caldwell et al., 2008). Since Dahl S rats exhibit increases in peripheral PICs (Huang et al., 2016), and PICs are important in regulating SNA and AVP release, we anticipated that these inflammatory cytokines may also be elevated in the brain, particularly in the PVN, and be involved in regulating the activity of the PVN neurons.

In this study, we demonstrated that HS diet induced significant increases in the mRNA levels of PVN PICs including *TNF-*α, *IL-6*, and *IL-1*β, as well as *Fra1*, a chronic neuronal activation marker, in Dahl S rats, but not in normal SD rats (Figure 2). ICV hypertonic sodium infusion to increase CSF [Na^+^] significantly upregulated mRNA transcripts of *TNF-*α, *IL-6, IL-1*β, and *Fra1* in the PVN of normal SD rats (Figure 6). Osmolarity control also significantly increased mRNA levels of TNF-α and showed an increase trend in IL-1β, but the increase extent was much less than that in hypertonic saline infused rats, indicating increased CSF [Na^+^] is a major drive promoting PICs and Fra1 expression. To further investigate whether the increased PICs is possibly involved in the regulation of sympathetic nerve activity, and/or neuropeptide AVP production and secretion, we further performed immunostaining to assess protein expression of PICs and Fra1 in three PVN subnuclei including VM and DC, two major autonomic-related PVN sub-regions which concentrated with pre-autonomic neurons; and LM subnucleus, a region that contains oxytocin and AVP magnocellular neurosecretory neurons. The results showed that in response to 5-week HS diet intake, a significant increase in TNF-α expression was found in the LM region; increased expression of IL-6 and Fra1 were observed in both LM and VM subnuclei in Dahl S rats. No significant changes were observed in the DC area for any of the three target proteins in Dahl S rats with HS diet compared to NS diet controls. Central administration of hypertonic saline dramatically increased expression of TNF-a, IL-6, and Fra1 (indicated by CTCF in Figures 7–9) although density of immunoreactive cells for different target protein varies (Figures 7–9). In summary, the immunostaining results demonstrate significant increases in PICs and Fra1 within the LM subnucleus, a region containing AVP neurosecretory neurons (Cruz et al., 2008; Biancardi et al., 2010) in both HS treated Dahl S rats and SD rats that received central administration of hypertonic saline. Interestingly, our recent published results showed that HS diet can dramatically increases AVP expression in the PVN of Dahl S rats (Huber et al., 2017). This evidence coupled with the evidence that PICs such as IL-1β and IL-6 can stimulate AVP secretion (Yasin et al., 1994; Chikanza et al., 2000) suggest that HS diet may increase plasma AVP through increasing PVN PICs expression. It is important to take into account that the neurons in the LM is not exclusively involved in neurohormones production and secretion, some neurons in this brain region also project to the RVLM and are probably involved in controlling SNA outflow (Fan et al., 2018). In addition, HS diet intake Dahl S rats and SD rats that received ICV infusion of hypertonic salt also presented increased IL-6 and Fra1 expression in the VM subnucleus, a key area concentrated with pre-autonomic neurons. Previous studies from other groups showed that microinjection of TNF-α into the PVN acutely increased lumbar and splanchnic SNA and mean arterial pressure (Bardgett et al., 2014). Intrahypothalamic perfusion with IL-1β stimulated AVP release in rats (Watanobe and Takebe, 1993). Therefore, increased PICs in PVN may contribute to elevation in sympathetic outflow through exciting the pre-autonomic neurons in the VM area and in AVP secretion through excitations of AVP producing neurons in the LM area.

One inconsistency we observed was that central administration of hypertonic salt also increased IL-6, TNF-α, and Fra1 expression in the PVN DC subnucleus, this increase was not found in the HS intake Dahl S rats. We do not know the exact reason for this. It may due to the higher sodium concentration used in the acute experiment. ICV infusion of 4 μmol of NaCI can increase CSF [Na^+^] by 10 mM while our results showed that HS diet treatment for 5-week increased CSF [Na^+^] by 5.5 mM in Dahl S rats. In addition, long term exposure to increased CSF [Na^+^] may trigger compensatory effect in the rats which may alter gene expression profiles in the PVN.

Finally, we investigated whether HS induced increase in PICs expression occurs in brain neurons. High salt (10 mM NaCl plus full culture medium) treatment in hypothalamic neurons isolated from neonatal SD rats resulted in significant increases in *TNF-*α and *IL-6*, as well as Fra1, but no difference was observed in *IL-1*β expression. In contrast, the expression of *IL1-*β was significantly increased in the PVN of high salt intake Dahl S rata as well as the normal SD rats that received hypertonic saline central administration. This difference may due to the following reasons: (1) The PVN is composed of many types of cells such as neurons, astrocytes and microglia. All three types of cells are able to produce cytokines (Sei et al., 1995), while microglia has been regarded as primary PICs resources (Kapoor et al., 2016a,b). The PICs produced from microglia can further regulate neuronal activity through binding to their receptors expressed on the membrane surface of neurons (Shi et al., 2010). Brain PICs have been well established to alter neuronal excitability through changes in ion channel receptor expression (Schafers and Sorkin, 2008) and/or gating properties/trafficking (Beattie et al., 2002). The communications between microglia and neurons can result in neuronal excitation. However, there is a lack in communication between different types of cells in brain primary neuronal cultures. (2) Astrocytes are also a key player in neuroinflammation. A recent study demonstrated that HS treatment can increase expression of PICs including TNFα, IL1β, and IL6 in cultured astrocytes (Deng et al., 2017). Therefore, the increased IL-β *in vivo* may be produced by astrocytes, again, the increased cytokines secreted from astrocytes can excite neurons by binding their receptors distributed in the neuronal cell membrane surface. (3) The PVN receives inputs from many other brain areas such as the circumventricular organs including subfornical nucleus (SFO) and organum vasculosum of lamina terminalis (OVLT). The SFO and OVLT are structures that lack of normal blood brain barriers and therefore can sense the changes in the systematic circulation such as plasma osmolarity changes and then pass that information into other brain regions including the hypothalamic PVN. This information may alter PVN gene expression profiles.

To summarize, our study indicates that a HS diet increases CSF sodium concentration. The increased CSF [Na+] subsequently upregulates PVN PICs expression, increases PVN neuronal activity and induces hypertension, in Dahl S rats. The increases in PIC and neuronal activity occur in both VM and LM subnuclei, two PVN regions involved in sympathetic outflow control and AVP production, respectively. The results suggest that a HS diet induces excessive PVN PICs production, which may through modulating sympathetic outflow and stimulating AVP-expression and secretion, contribute to the initiation and development of SSHTN.

## Author contributions

EJ and ZS conception and design of experiments; EJ, YF, AC, RL, MH, and TH performed experiments; EJ, YF, and ZS analyzed data; EJ and ZS interpreted results of experiments; YF prepared figures; AC drafted manuscript; EJ, JY, Q-HC, and ZS edited and revised manuscript; EJ and ZS approved final version of manuscript.

### Conflict of interest statement

The authors declare that the research was conducted in the absence of any commercial or financial relationships that could be construed as a potential conflict of interest.

## Abbreviations

AVP: vasopressin
CSF: cerebrospinal fluid
Dahl S: Dahl salt sensitive
HS: high salt
IL-6: Interleukin-6
IL-1β: Interleukin-1 beta
ICV: intracerebroventricular
MAP: mean arterial pressure
NS: normal salt
PVN: paraventricular nucleus
PICs: proinflammatory cytokines
OVLT: organum vasculosum of lamina terminalis
SD: Sprague Dawley
Sodium concentration: [Na^+^]
SSHTN: salt sensitive hypertension
SFO: subfornical nucleus
TNF-α: Tumor necrosis factor alpha.
